# Supplementary material for: Global variation in the fraction of leaf nitrogen allocated to photosynthesis
Source: Nat Commun. 2021 Aug 11;12:4866. doi: 10.1038/s41467-021-25163-9 (PMC8358060; doi:10.1038/s41467-021-25163-9)
Supplement: Supplementary file 1 — Supplementary Information [file 41467_2021_25163_MOESM1_ESM.docx]

**Supplementary materials**


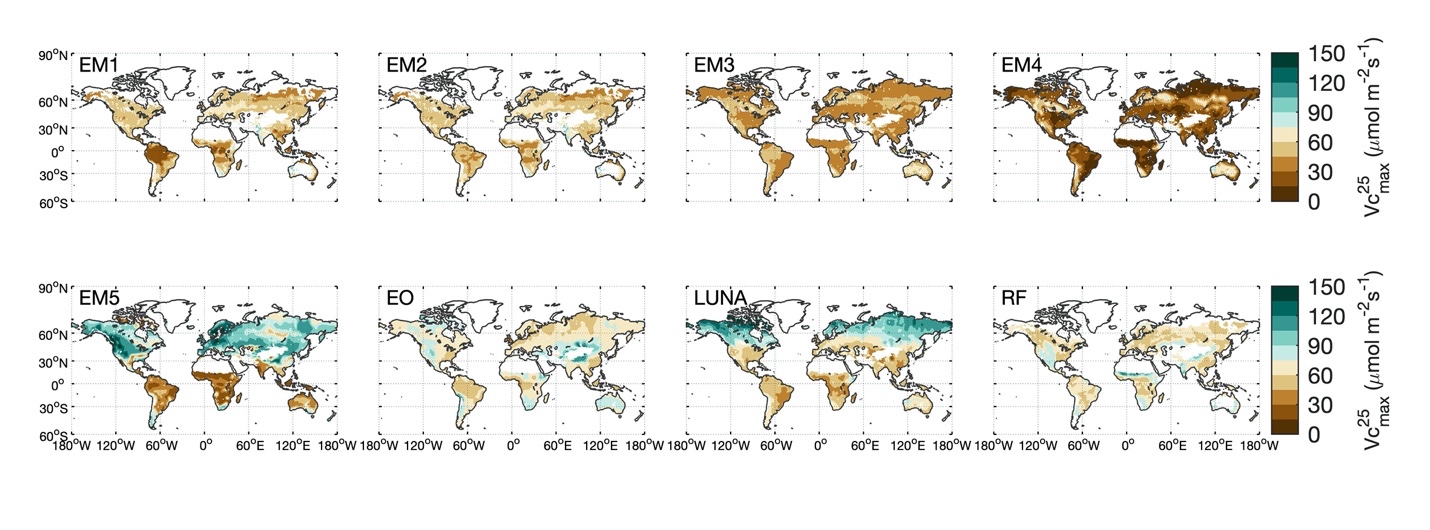


**Supplementary Figure 1.** **Global** $\boldsymbol{V}_{\boldsymbol{c}_{\boldsymbol{max}}}^{\boldsymbol{25}}$ **estimated by competing** $\boldsymbol{V}_{\boldsymbol{c}_{\boldsymbol{max}}}^{\boldsymbol{25}}$ **models and random forest (RF).** The models include five empirical $V_{c_{max}}^{25}$ models (EM1 to EM5) and two optimal $V_{c_{max}}^{25}$ (EO and LUNA). The maps were created by the authors using a Matlab package M_Map (see Code Availability Statement).


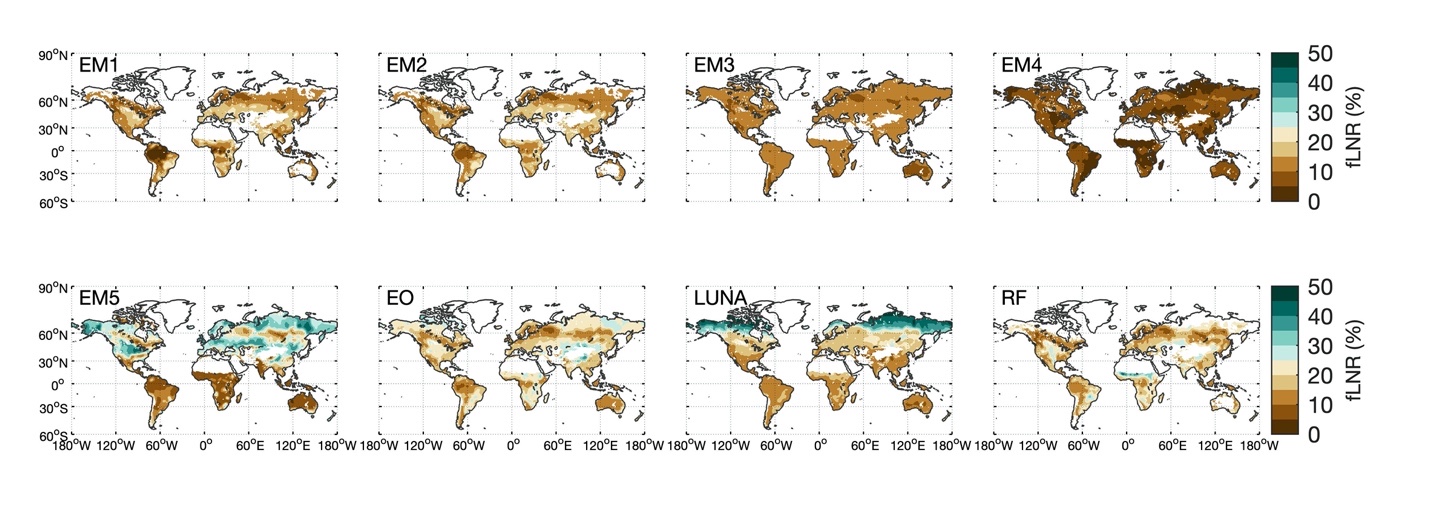


**Supplementary Figure 2.** **Global fLNR** **implied by competing** $\boldsymbol{V}_{\boldsymbol{c}_{\boldsymbol{max}}}^{\boldsymbol{25}}$ **models and the random forest (RF)** $\boldsymbol{V}_{\boldsymbol{c}_{\boldsymbol{max}}}^{\boldsymbol{25}}$**.** The models include five empirical $V_{c_{max}}^{25}$ models (EM1 to EM5) and two optimal $V_{c_{max}}^{25}$ models (EO and LUNA). The maps were created by the authors using a Matlab package M_Map (see Code Availability Statement).


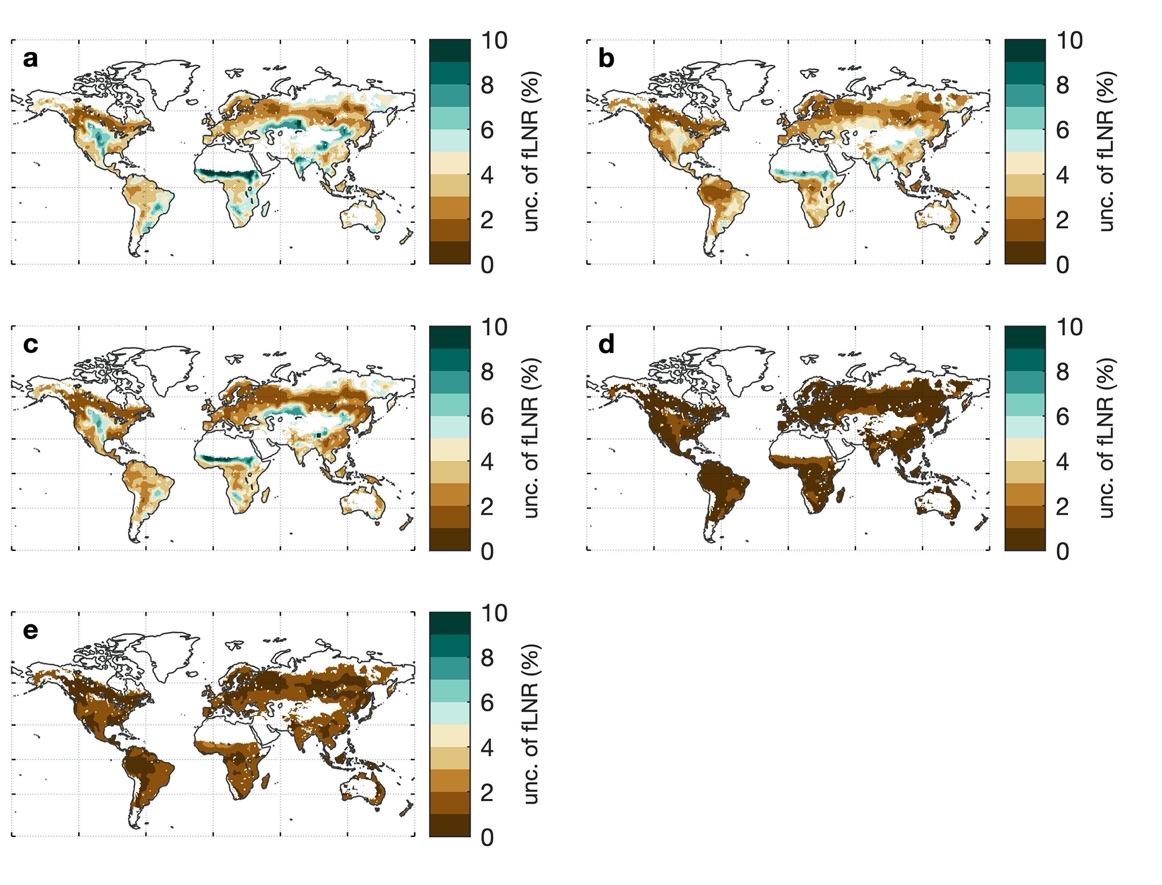


**Supplementary Figure 3. Uncertainty of global fLNR produced by a random forest model (RF).** (a) total uncertainty; (b) uncertainty incurred by RF $V_{c_{max}}^{25}$; (c) uncertainty incurred by LNC; (d) uncertainty incurred by α^25^; (e) uncertainty incurred by fNR. The maps were created by the authors using a Matlab package M_Map (see Code Availability Statement).


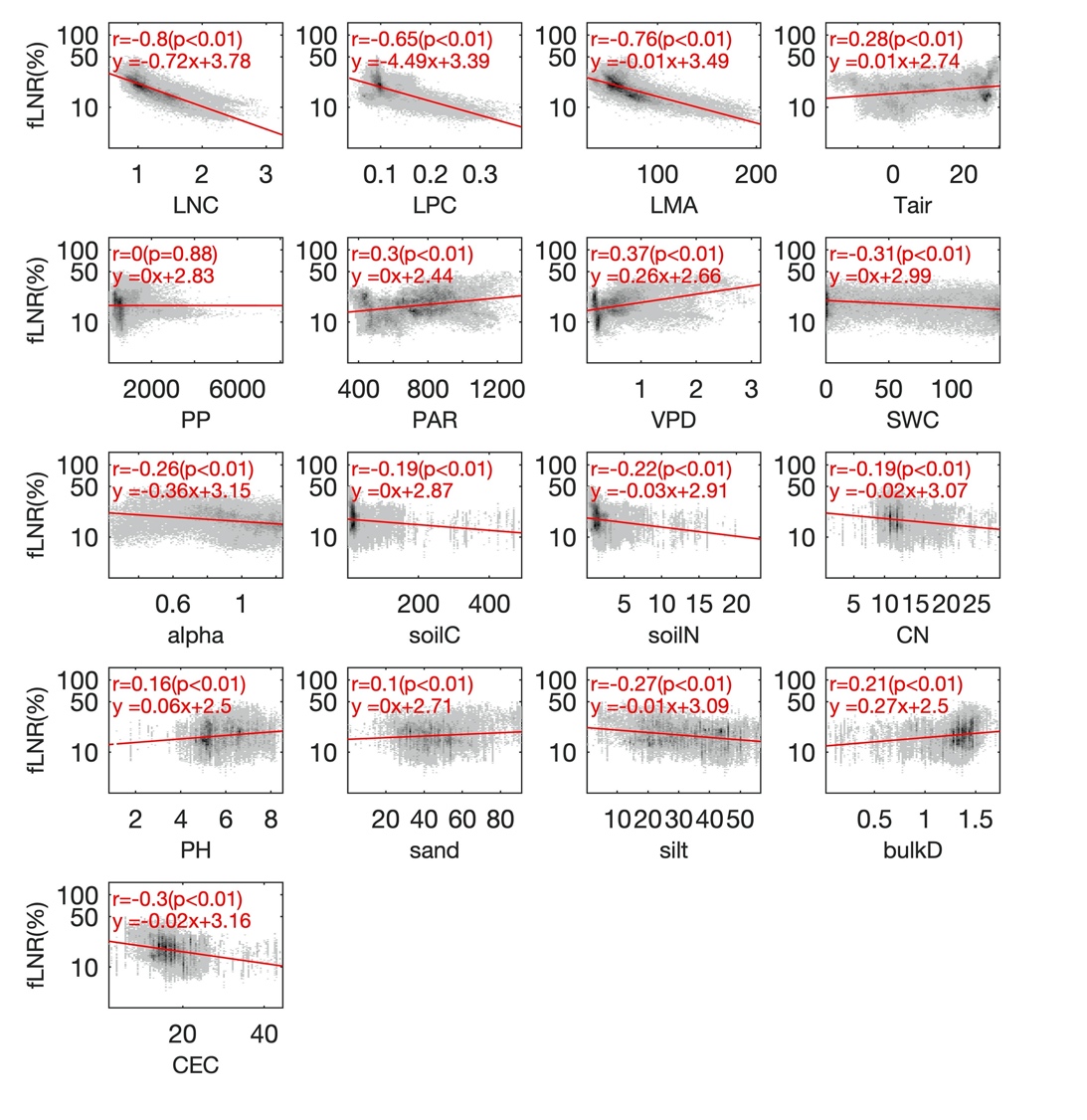


**Supplementary Figure 4.** Univariate regression of log-transformed fLNR to leaf traits, climate and soil. The base of log transformation is the natural constant e. Leaf traits include leaf nitrogen content (LNC), leaf phosphorus content (LPC) and leaf mass per area (LMA); climate variables includes annual mean air temperature (Tair), precipitation (PP), photosynthetic active radiation (PAR), vapor pressure deficit (VPD), soil water content (SWC) and the ratio of evapotranspiration to potential evapotranspiration (alpha); soil variables includes soil organic carbon content (soilC), total nitrogen content (soilN), CN ratio, pH, the percentage of sand (sand), the percentage of silt (silt), bulk density (bulkD) and cation exchange capacity (CEC).


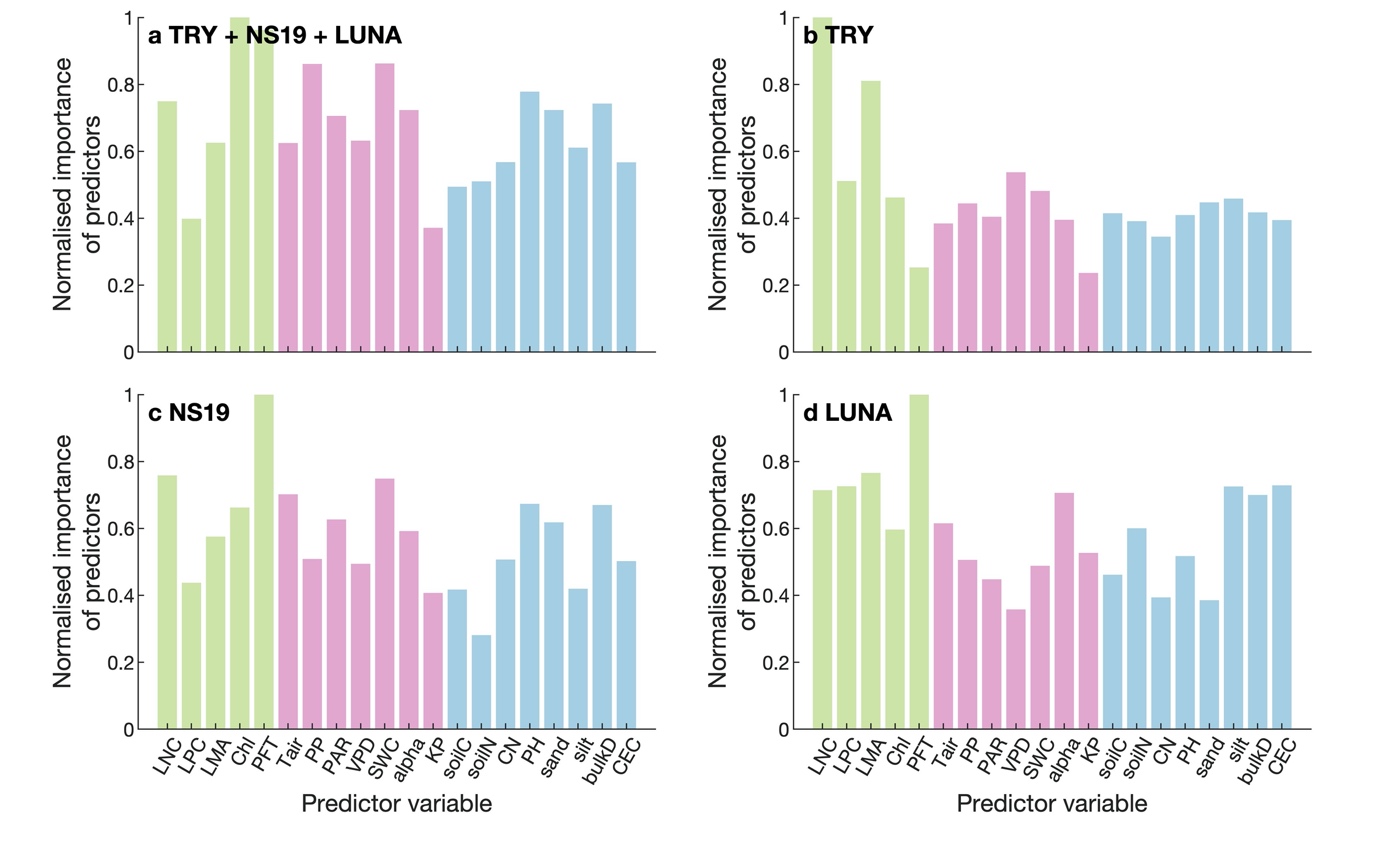


**Supplementary Figure 5. Normalized importance of variables for different** $\boldsymbol{V}_{\boldsymbol{c}_{\boldsymbol{max}}}^{\boldsymbol{25}}$ **datasets.** We amass $V_{c_{max}}^{25}$ observations from three sources: TRY database, NS19 dataset and training data compiled for LUNA model. The importance of 20 variables from three groups -- leaf and canopy traits (green), climate (red) and soil (blue) – are evaluated using 200 bagged decision trees. The 20 variables include: leaf nitrogen content (LNC), leaf phosphorus content (LPC), leaf mass per area (LMA), leaf chlorophyll content (Chl), Plant functional type (PFT), annual mean air temperature (Tair), precipitation (PP), photosynthetic active radiation (PAR), vapor pressure deficit (VPD), soil water content (SWC) and the ratio of evapotranspiration to potential evapotranspiration (alpha), Koeppen climate classification, soil organic carbon content (soilC), total nitrogen content (soilN), CN ratio, pH, the percentage of sand (sand), the percentage of silt (silt), bulk density (bulkD) and cation exchange capacity (CEC).

**
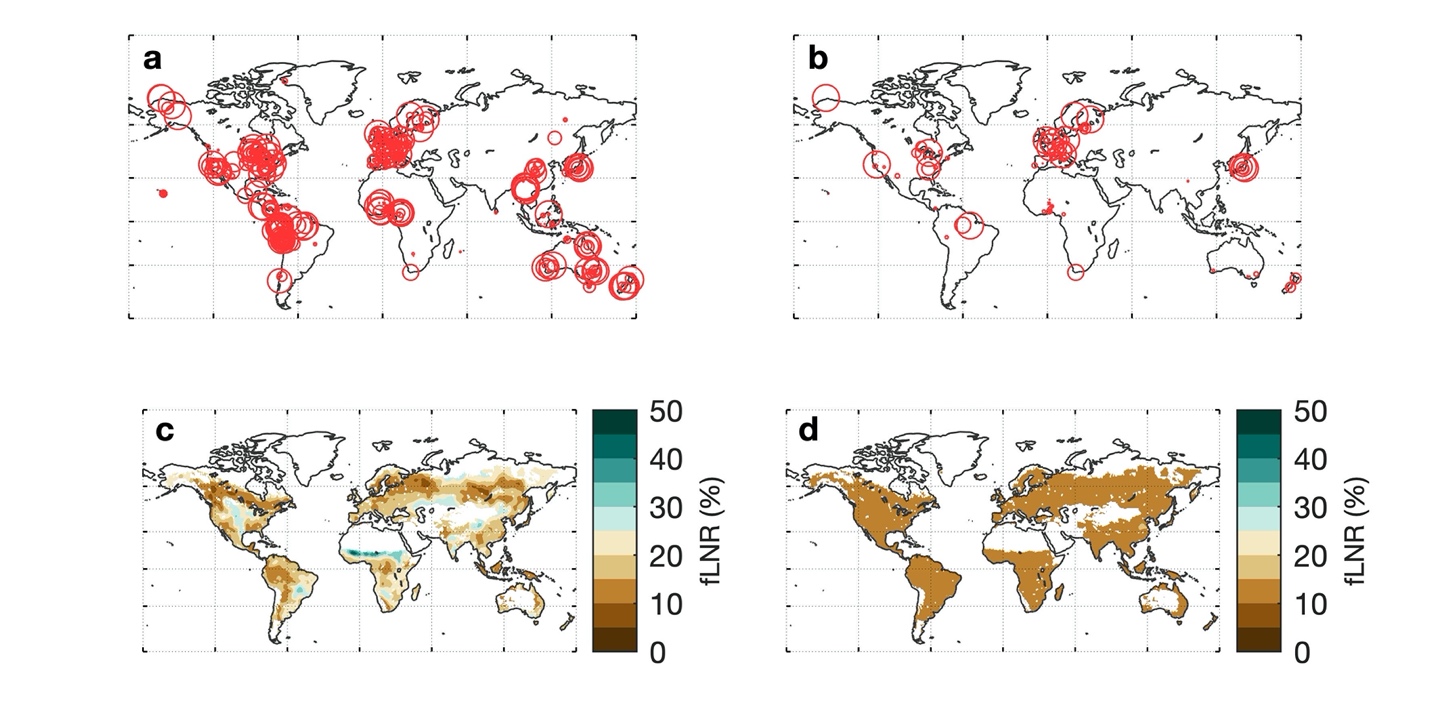
**

**Supplementary Figure 6. Distribution of** $\boldsymbol{V}_{\boldsymbol{c}_{\boldsymbol{max}}}^{\boldsymbol{25}}$ **and fLNR observations.** (a)$V_{c_{max}}^{25}$ and (b) fLNR. The fLNR observations were acquired by applying Equation (1) to concurrent observations of $V_{c_{max}}^{25}$ and LNC at sites. The size of the circles indicates the number of observations. The largest circle indicates > = 40 obs. (c) The global fLNR map presented in this study, obtained based on RF $V_{c_{max}}^{25}$; (d) the fLNR map directly trained from fLNR observations in (b). The maps were created by the authors using a Matlab package M_Map (see Code Availability Statement).


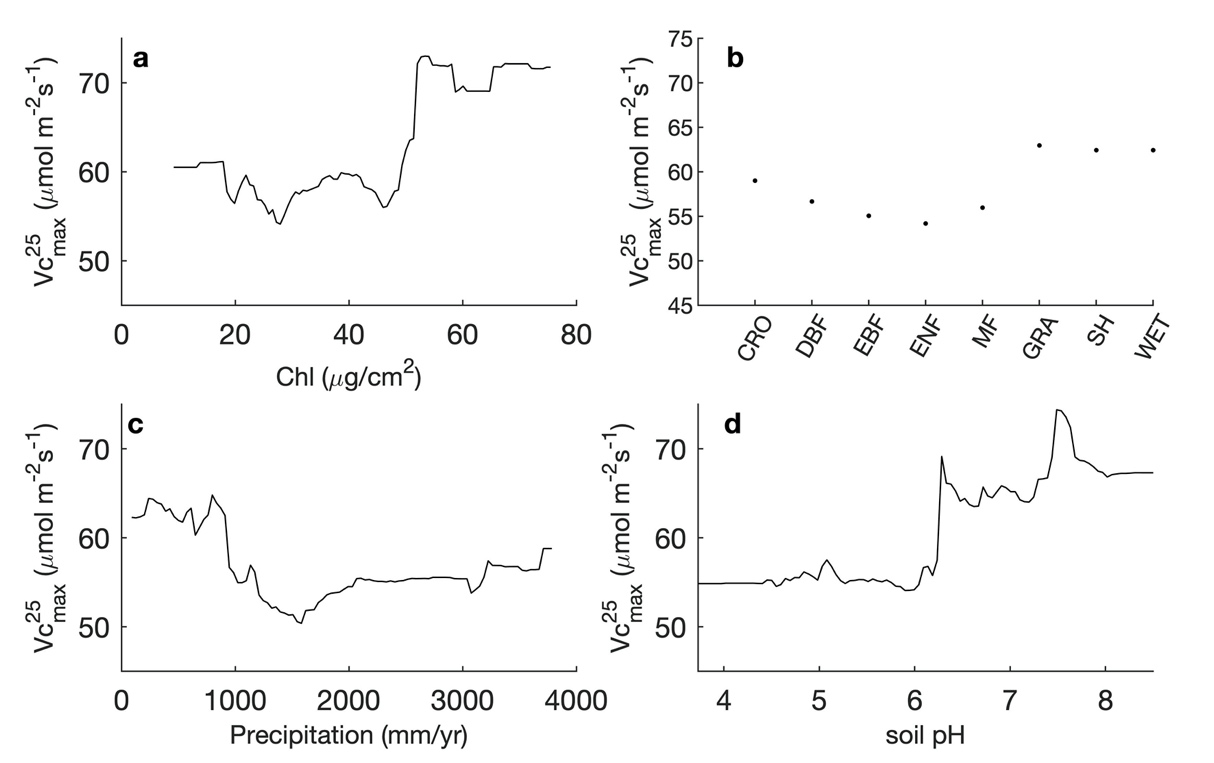


**Supplementary Figure 7. The partial dependence of** $\boldsymbol{V}_{\boldsymbol{c}_{\boldsymbol{max}}}^{\boldsymbol{25}}$ **on predictors of the random forest model.** The dependences of $V_{c_{max}}^{25}$ on (a) leaf chlorophyll content (Chl), (b) plant functional types (PFTs), (c) mean annual precipitation and (d) soil pH.


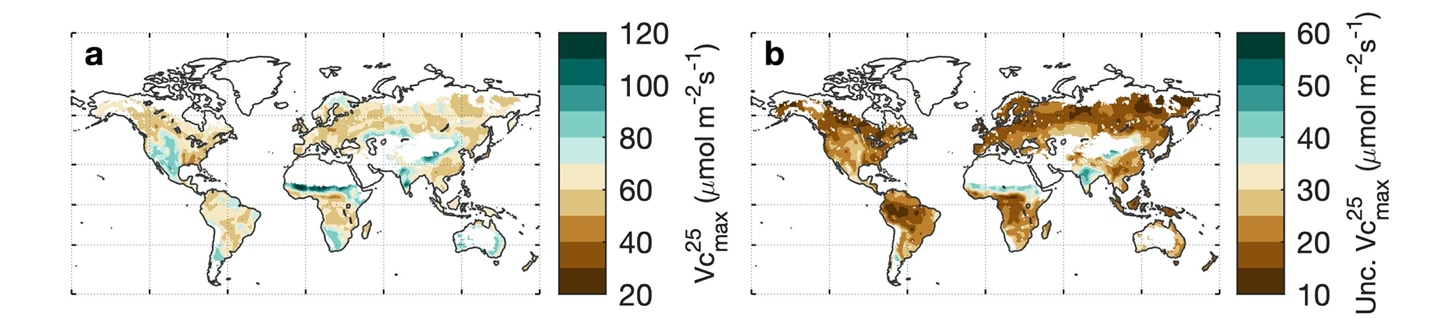


**Supplementary Figure 8. The** $\boldsymbol{V}_{\boldsymbol{c}_{\boldsymbol{max}}}^{\boldsymbol{25}}$ **estimated by the random forest model (RF) and its uncertainty.** (a) $V_{c_{max}}^{25}$ and (b) the uncertainty of $V_{c_{max}}^{25}$, indicated by one standard deviation of the estimates from 200 bagged RF trees. The maps were created by the authors using a Matlab package M_Map (see Code Availability Statement).


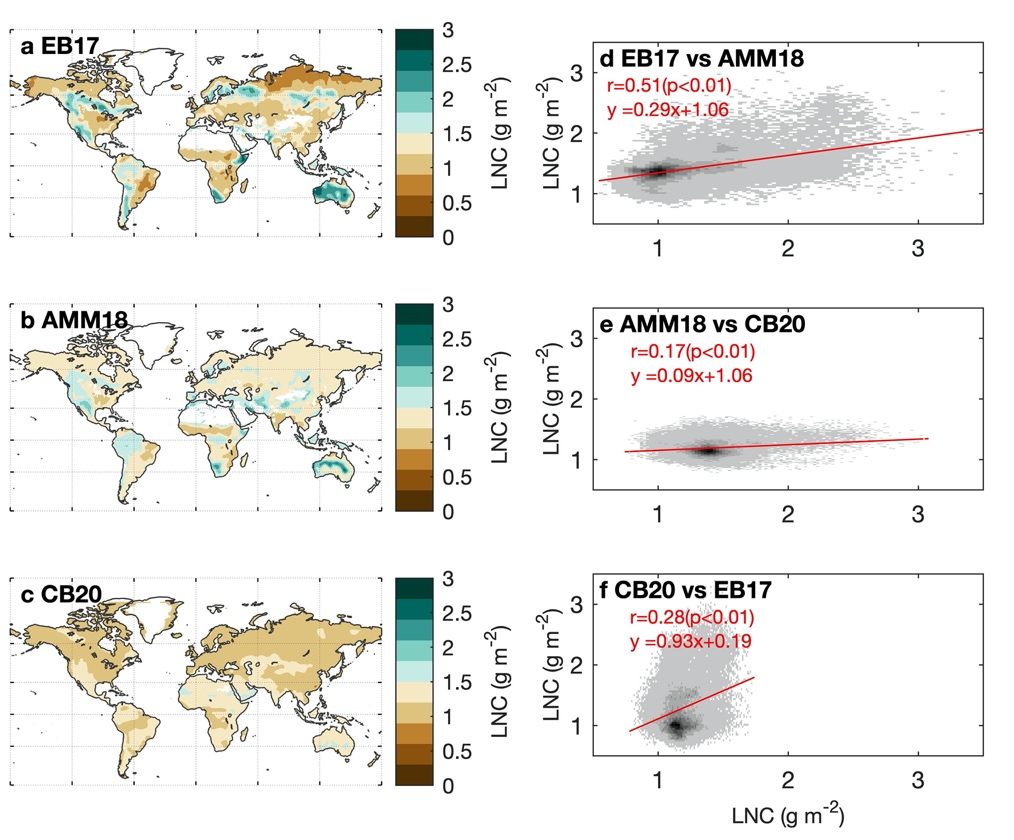


**Supplementary Figure 9.** **Comparison of three published global leaf nitrogen content (LNC) maps** ^28,31,33^. (a,b,c) the spatial variation of LNC and (d,e,f) the spatial correlations between the LNC maps. The accesses to different LNC datasets are available in the Data availability statement. The maps were created by the authors using a Matlab package M_Map (see Code Availability Statement).


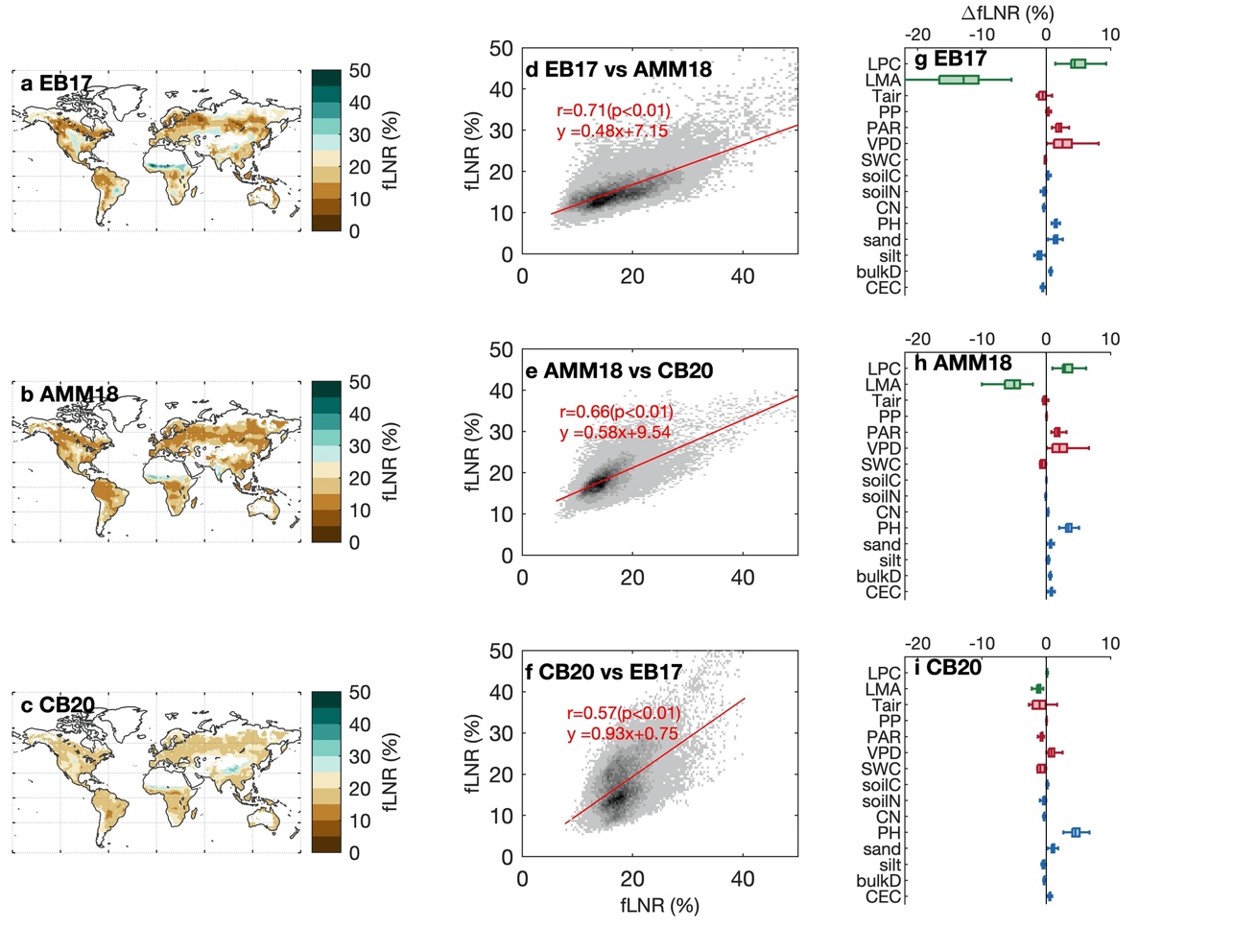


**Supplementary Figure 10.** **Three global fLNR maps estimated using alternative leaf nitrogen content maps** ^28,31,33^. (a,b,c) the spatial variation of fLNR, (d,e,f) the spatial correlations between the fLNR maps and (g,h,i) the responses of fLNR to environmental variables. The changes in fLNR (%) attributed to different variables. For each box plot, the centre line indicates the median, the box indicates the upper and lower quartiles and the whiskers indicate 1.5 times the interquartile range away from the top or bottom of the box (n = 39147). The maps were created by the authors using a Matlab package M_Map (see Code Availability Statement).


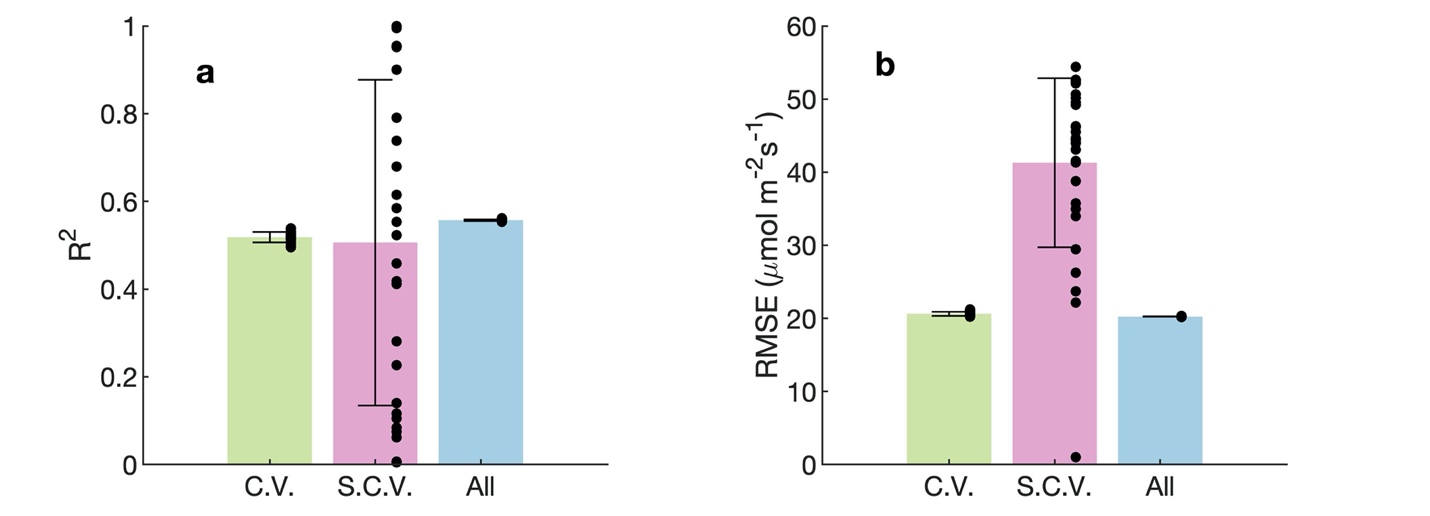


**Supplementary Figure 11.** The validation of the RF $V_{c_{max}}^{25}$. ‘C.V.’ indicates conventional cross-validation, ‘S.C.V.’ indicates spatial cross-validation, ‘All’ indicates using all available samples for validation. (a) R^2^ of validation; (b) RMSE of validation. Each type of validation was conducted 30 times (n = 30). The heights of the bars indicate the mean values, and the error bars indicate one standard deviation of validation runs.


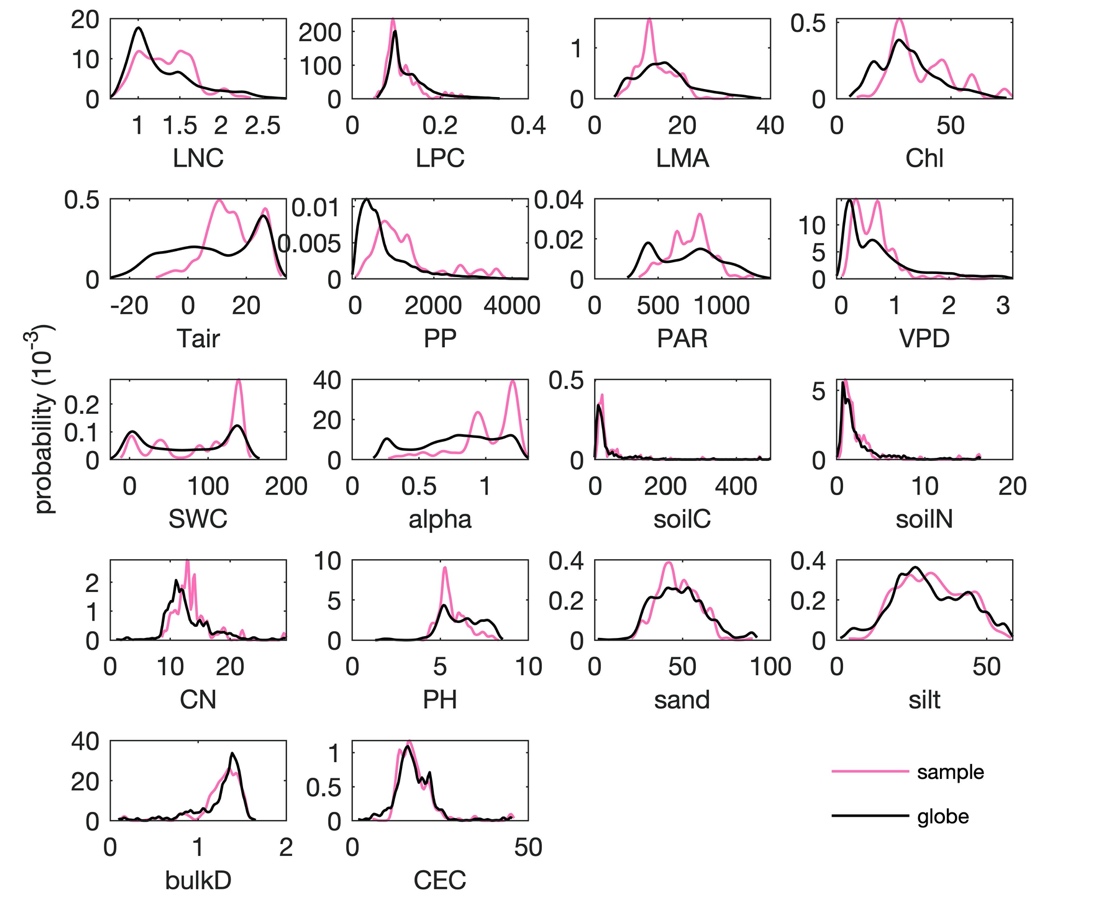


**Supplementary Figure 12.** The representativeness of samples for global extrapolation.

**Supplementary Table 1.** The decomposition of three groups of variables (i.e., leaf traits, climate and soil) using principal component analysis.

| Type |  | PC1 | PC2 | PC3 |
| --- | --- | --- | --- | --- |
| Leaf | Variance explained (%) | 83.4 | 14.5 | 3.1 |
|  | Coefficient of variables |  |  |  |
|  | LNC | 0.59 | -0.46 | -0.66 |
|  | LPC | 0.52 | 0.84 | -0.11 |
|  | LMA | 0.61 | -0.28 | 0.74 |
| Climate | Variance explained (%) | 57.3 | 31.7 | 5.0 |
|  | Coefficient of variables |  |  |  |
|  | Temperature | 0.38 | 0.48 | 0.07 |
|  | Precipitation | -0.11 | 0.66 | -0.42 |
|  | PAR | 0.49 | 0.24 | 0.02 |
|  | VPD | 0.50 | 0.10 | 0.53 |
|  | SWC | -0.45 | 0.23 | 0.73 |
|  | Aridity | -0.38 | 0.45 | 0.04 |
| Soil | Variance explained (%) | 60.0 | 16.0 | 12.7 |
|  | Coefficient of variables |  |  |  |
|  | Soil C | 0.42 | 0.14 | 0.09 |
|  | Soil N | 0.44 | 0.05 | 0.01 |
|  | CN | 0.37 | 0.28 | 0.24 |
|  | PH | -0.21 | -0.28 | 0.79 |
|  | Sand | -0.22 | 0.66 | 0.38 |
|  | Silt | 0.29 | -0.59 | 0.12 |
|  | Bulk density | -0.39 | -0.20 | 0.17 |
|  | CEC | 0.40 | 0.00 | 0.37 |

**Supplementary Table 2.** The empirical equations to derive fraction of nitrogen invested in RuBisCO (fLNR) from key biological and environmental factors. These key factors include leaf mass per area (LMA; unit: g/m^2^), leaf phosphorus content (LPC; unit: g/m^2^), vapor pressure deficit (VPD; unit: kPa), photosynthetic active radiation (PAR; unit: μmol/m^2^/s), soil pH and soil sand percentage (Sand; unit: %).

| fLNR(%) | LMA | LPC | VPD | PAR | pH | Sand | Intercept |
| --- | --- | --- | --- | --- | --- | --- | --- |
| Overall | -0.19 | 42.2 | 4.76 | 0.0026 | 0.25 | 0.032 | 2.4 |
| CRO | -0.25 | -10.8 | 4.38 | 0.0047 | 0.22 | 0.033 | 11.0 |
| DBF | -0.15 | 23.0 | 4.41 | 0.0017 | 0.19 | 0.031 | 3.6 |
| EBF | -0.24 | 72.8 | 2.26 | 0.0039 | 0.14 | 0.030 | -0.9 |
| ENF | -0.09 | 8.3 | 3.55 | -0.0015 | 0.26 | 0.008 | 2.3 |
| MF | -0.20 | 43.3 | 5.73 | -0.0012 | 0.13 | 0.033 | 4.7 |
| GRA | -0.19 | -24.5 | 3.98 | 0.0038 | 0.18 | 0.034 | 9.8 |
| SH | -0.20 | -37.1 | 2.93 | 0.0049 | 0.30 | 0.033 | 10.6 |
| WET | -0.15 | 22.7 | 4.63 | 0.0018 | 0.22 | 0.035 | 0.4 |

**Supplementary Table 3.** Evaluation of LNCm and SLA of the three products: EB17 ^28^, AMM18 ^33^, CB20 ^31^

| Products | LNCm (mass-based; mg/g) | | SLA (m^2^/kg) | |
| --- | --- | --- | --- | --- |
|  | R^2^ or pseudo R^2^ | RMSE or RMPSE | R^2^ or pseudo R^2^ | RMSE or RMPSE |
| EB17 | 0.548 | 6.18 | 0.602 | 6.13 |
| AMM18 | 0.539 | 2.30 | 0.582 | 3.19 |
| CB20 | < 0.3 |  | < 0.2 |  |
